# Military Benevolent Fund

**Published:** 1901-04-27

**Authors:** 


### The Institutional Workshop.

### MILITARY BENEVOLENT FUND.

### A NEW MANAGEMENT SCHEME.

As the result of a motion made before Mr. Justice Kekewich in the Chancery Division of the High Court of Justice last week, an order was made which provides for the temporary management of the Royal Military Benevolent Fund, and prepares the way for a permanent arrangement. The motion made in the proceedings at the instance of Mrs. Ellis Williams, secretary and treasurer to the charity, the Attorney-General being respondent, asked that, pending the settlement by the Court of a scheme for the due administration of the funds, the plaintiff, on giving security, might be appointed to receive the income, and thereout to pay the annuities granted by the fund and the expenses incident to the carrying on of the school, which forms part of the charity, of the general working and maintenance of the charity, and carrying on of its business.

Mr. Renshaw, K.C., and Mr. Church appeared in support of the motion, and the Attorney-General and Mr. R. J. Parker represented the fund.

Mr. Renshaw stated that, having had an opportunity of consultation with the Attorney-General, he thought they would be able to arrange, subject to his lordship's sanction, a form of judgment. They had agreed to treat the motion as a trial of the action and to take a judgment in the action which was brought by Mrs. Ellis Williams against the Attorney-General. It was asked that the funds and the property of the charity might be administered under the Court, and pending a settlement of such a scheme that a proper person might be appointed as receiver and manager. His lordship might have seen in the public press some reference to this charity in the course of last year. He did not propose—and he was sure the Attorney-General did not wish—to go into the matters of controversy between the parties. The charity was founded in the year 1874 by Mrs. Ellis, who was then Miss Davis, the daughter of an officer of the Army, for the purpose of providing annuities for the widows and unmarried daughters of officers of the Army who were in needy circumstances. At a later date it was proposed that a home should be instituted for the purpose of providing a residence for the annuitants, and money was collected for that purpose. That scheme fell through, partly because some annuitants did not like to live in a home and partly because others desired to bring relations and friends with them. Without going into details, a school was in place of the home, established for the education of the daughters of deceased officers, although their mothers might be living. The result was that now there was a fund representing investments of £11,750, which stood invested in various securities. In consequence of certain matters, with which he did not propose to deal, it was necessary to resort to the court. Before the issue of the writ there was some communication with the Charity Commissioners with regard to whether it was necessary to get their assent to any scheme, but in order to avoid any difficulty the Attorney-General had issued another writ that morning, asking practically for the same arrangement as was asked by the plaintiff. They had, he was told, not received the consent of the Charity Commissioners, but it had not been refused.

The Attorney-General said the second writ had not yet been served, but there was no doubt service would be accepted, and any order made in the one case would be treated as an order made in the other. He was told one of the trustees was in South Africa, and in regard to him there might be some delay.

Mr. Renshaw, proceeding, said it was desirable that they should have some immediate order, because there were payments in respect of annuities which were now overdue, and expenses in connection with the school which ought to be paid promptly. In these circumstances, his learned friend and he had come to the conclusion that what they ought to suggest to his lordship was that there should be a form of judgment, and they proposed that the hearing of the motion should be treated as a trial of both actions, and that then they should take an order to administer the funds. For that purpose a scheme would have to be sanctioned and settled by the Court. They next proposed that, pending the settlement of such scheme, Sir Henry Burdett should be appointed to receive the income of the society, and thereout, to pay the annuities granted by the charity and the expenses of the school. They proposed, further, that the charity, including the school, should be managed under the direction of the Court, pending the settlement of a scheme. The Attorney-General would shortly apply for the appointment of some controlling committee. In the meantime, the charity would be carried on as it had hitherto been. Then they proposed that the necessary inquiry as to the funds would take place under joint action.

The Attorney-General said he was glad that they had been able to arrange an order in the terms which had been mentioned, otherwise a number of persons to whom these annuities were of the greatest possible value would be greatly inconvenienced. This order would enable Sir Henry Burdett to act at once and pay these annuities.

Mr. Justice Kekewich thought there might be a difficulty in making the order, one of the trustees being in South Africa, unless the gentleman's solicitor was empowered to act for him in the matter.

The Attorney-General explained that in regard to the trustee in question, Colonel Mount-Stephen, he was not a trustee for the fund generally, but certain stock of a comparatively small amount was standing in his name.

Mr. Justice Kekewich said he was anxious to assist counsel. If nobody could act for the gentleman in the matter, then he could be struck out of the action altogether, and the result would merely be that Sir Henry Burdett would not have control of that small fund.

The Attorney-General said that would enable an order to be made which would empower Sir Henry Burdett, of whose qualifications he need say nothing, to act at once, even without the formality of security being gone through. With regard to the business of the scheme, pending a settlement, he desired that the receiver should be able to act from that day. At an early day he should apply to the Court to appoint a committee to take control of the charity, which meantime would be under the direction of the Court. He hoped the result would be that at a very early day this charity would be put on a basis which would be satisfactory to everyone concerned.

Mr. Justice Kekewich: There is no reason why it should not be.

The order was then made in the terms suggested by counsel.

